# Etiology and Management of Peristomal Pseudoepitheliomatous Hyperplasia

**DOI:** 10.7759/cureus.20196

**Published:** 2021-12-06

**Authors:** Farah Alslaim, Farah Al Farajat, Hossam S Alslaim, Peter Drevets, Barry Jenkins

**Affiliations:** 1 General Surgery, Jordan University of Science and Technology, Irbid, JOR; 2 General Surgery, Augusta University Medical College of Georgia, Augusta, USA; 3 Colorectal Surgery, University Hospital Health System, Augusta, USA

**Keywords:** pseudoepitheliomatous hyperplasia, stoma revision, enterocutaneous fistula, parastomal infection, stoma related complications

## Abstract

This is a report of a 48-year-old male patient who presented with worsening peristomal dermatitis. He proceeded to form a nodular hyperplastic overgrowth that was proven to be pseudoepitheliomatous hyperplasia on histological examination. On surgical exploration, he was found to have an apparent peristomal enterocutaneous fistula propagating this hyperplastic growth. This report reviews the etiology and management of peristomal complications with special attention to pseudoepitheliomatous hyperplasia.

## Introduction

The creation of ileostomies and colostomies is commonly performed for patients with intestinal and colonic diseases. They can be end stomas or diverting loop stomas and can either be permanent or temporary. Frequently, the decision to create a stoma depends on intraoperative findings so counseling the patients on this possibility preoperatively is paramount. These intestinal stomas are not without complications, and they create a significant strain on the patient’s lifestyle [1].

Beyond the physiological consequences of intestinal stomas, patients also undergo psychological distress due to altered body image and reduced personal hygiene associated with leakage [2]. The effects of these stomas are surreal: in a survey of 391 patients with a stoma, 80% reported changes in lifestyle and 40% reported impaired sexual activity. This has led to social distancing and a lack of self-confidence [2].

Long-term complication rates of 58% in colostomies and up to 76% in ileostomies have been reported [1]. The association between stoma complications and mortality has also been notable.

Stomal complications include parastomal hernia, dehydration and electrolyte imbalance, stomal prolapse or retraction, necrosis, and peristomal skin complications. The most common of these are parastomal hernias [3]. the incidence of these complications appears to have undergone little change despite the advances in surgical and postoperative care.

Peristomal skin conditions are reported in 18%-55% of patients with stomas [3]. These conditions can be attributed to one of four etiologies: mechanical, allergic, infectious, and chemical.

Mechanical injuries are associated with difficult-to-fit stomas. This is frequent in obese patients and patients who undergo an emergent procedure with poor siting. Retraction and prolapse also predispose to ill-fitting appliances. Ill-fitting appliances can create pressure on the skin and cause damage. Adequate preoperative assessment and meticulous attention to education for appliance placement technique is crucial [4].

Allergic contact dermatitis as a reaction to the appliance material has been described and typically presents as erythematous skin with vesicles. A change of appliance material or, if not feasible, a combination of antihistamine and topical steroids are helpful in such a case [3].

Infections can develop in moist, warm peristomal skin. The most common is cutaneous candidiasis presenting as white shiny pustules [5]. Topical antifungals are effective in these cases.

Chemical irritation can be caused by exposure of the skin to stoma output. This can be the result of a large opening in the stoma wafer or an ill-fitted appliance. During the patient’s life, intestinal stomas undergo size changes related to swelling, retraction, or prolapse. As such, fitting the appliance should be a dynamic process that accommodates those changes [6]. Failure to do so can lead to chronic contact dermatitis and skin erosions caused by chemical exposure. Some providers might feel compelled to create a larger wafer opening to alleviate this irritation, serving only to worsen the condition [7]. Prolonged irritation and exposure can cause a chronic overgrowth of pseudo-verrucous lesions. Those can make adequate fitting and pouching a challenging process, serving to create a vicious cycle [3].

A unique etiology of irritation is an aberrant peristomal fistula resulting in constant exposure of the peristomal skin to effluents. Herein, we present a patient with peristomal chemical skin irritation resulting from a peristomal fistula that has worsened to the point of requiring surgical intervention.

## Case presentation

**Figure 1:**
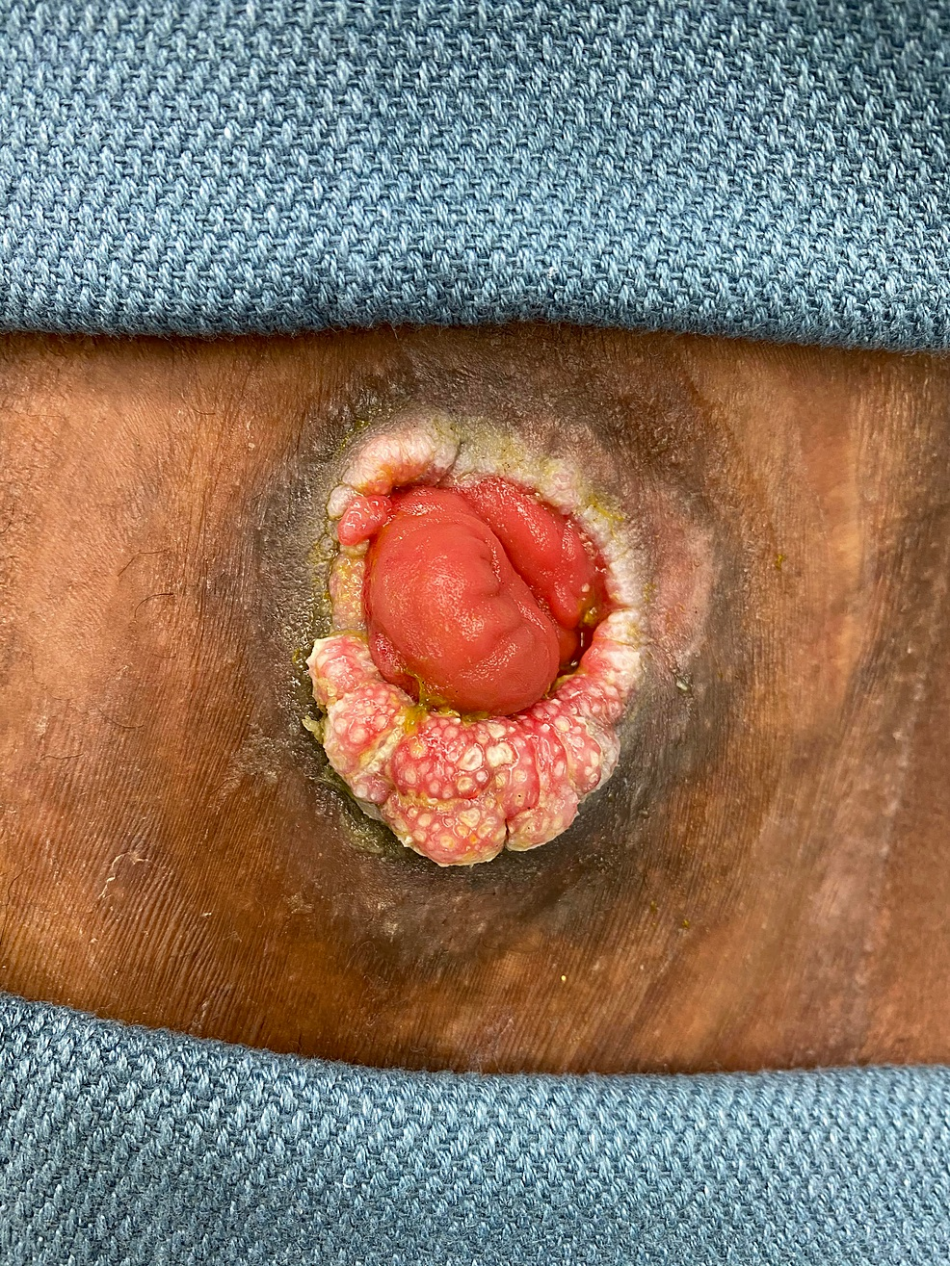
Peristomal skin growth Preoperative image of peristomal skin growth and irritation. Note the verrucous character of the lesion with crusted edges.

**Figure 2:**
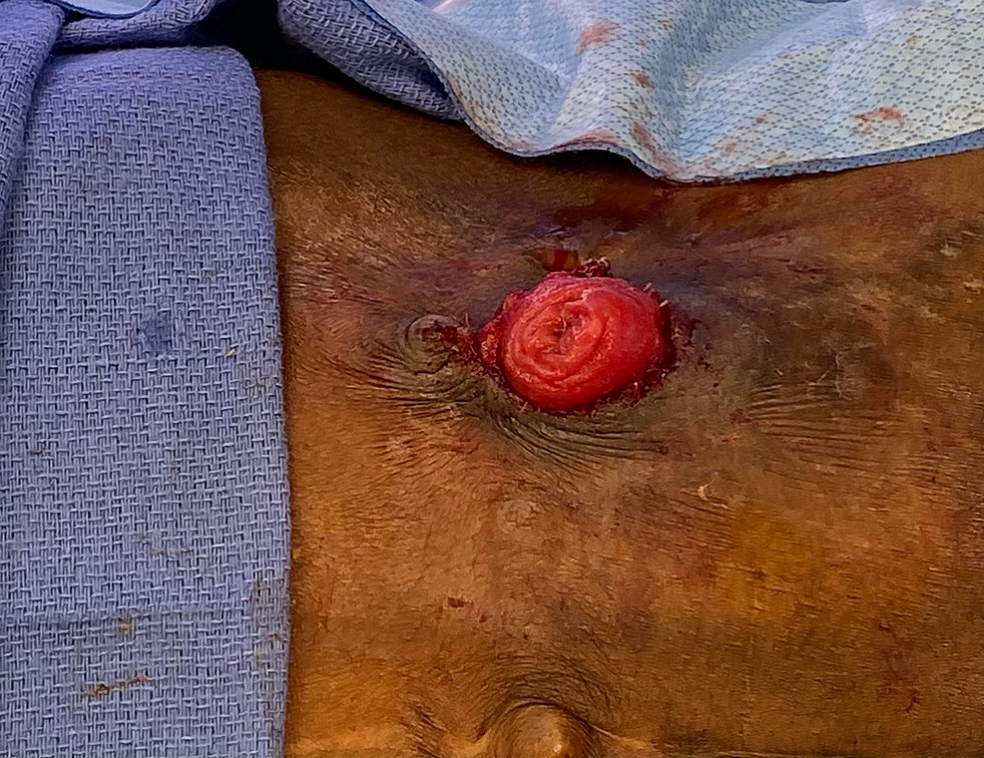
Post-revision Postoperative image showing the stoma after resection of the skin growth.

This is a 48-year-old male patient with a past medical history of end-stage renal disease. He was previously on peritoneal dialysis and failed two previous cadaver kidney transplants. He has been on hemodialysis since then. He has a history of recurrent small bowel obstruction that has previously required extensive adhesiolysis and resection of a large segment of his small bowel with a diverting loop ileostomy. For over three months, the patient has experienced progressive growth around his stoma as depicted in Figure 1. This growth is on the posterior aspect of his stoma and is associated with difficult pouching of the stoma, irritation, and drainage. Physical examination was consistent with pseudoverrucous characteristics to these lesions. The patient reported that he felt an intermittent draining sinus adjacent to his stoma but was not able to express any fluid on compression. Nonoperative management to include different stoma appliances and topical ointments was not successful to reverse the growth of this lesion. After two years of failed nonoperative management, he was referred to our facility for evaluation and possible intervention. The description of symptoms was concerning for an aberrant peristomal fistula. He underwent operative excision and revision of his stoma to address this growth. The skin surrounding the stoma was excised and sent for pathology. Intraoperatively, an apparent fistula in the right upper quadrant of the stoma was found, corroborating the patient's description of a draining sinus. This appeared to be from a very small enterotomy just below the skin level that was decompressing at the mucocutaneous junction. The patient was discharged on postoperative Day 2 without any complaints. Figure 2 depicts his stoma after the operation. Pathology reports showed pseudoepitheliomatous hyperplasia with hyperkeratosis and parakeratosis. The patient was seen for follow-up two weeks from his surgery and reports resolution of his complaints.

## Discussion

Pseudoepitheliomatous hyperplasia (PEH) is a benign skin condition characterized by hyperplastic growth of the epidermis [8]. It presents as well-demarcated plaques with nodules and skin crusting. PEH is associated with infectious, neoplastic, and inflammatory processes. They can present in the context of fungal and mycobacterial infections [8]. They are also associated with basal cell carcinoma, lymphomas, and can confound the diagnosis of melanocytic lesions. They can be misdiagnosed as squamous cell carcinomas and lead to patients being subjected to unnecessary surgeries [9]. More related to our topic, chronic inflammation and irritation can facilitate the growth of PEH. Such inflammatory conditions include lichen sclerosis, lichen simplex, pemphigus vegetans, and pemphigoid [10-11].

The presentation of pseudoverrucous lesions in the context of peristomal skin irritation has been well-documented by Stelton et al. [12]. They are thought to be hypertrophic “wart-like” lesions resulting from exposure to stoma effluent over a prolonged time course. Adequate stoma fitting, with minimum peristomal skin exposure, is believed to be helpful [12]. This patient presents a unique case of development of PEH as a result of an aberrant peristomal fistula. This creates a scenario that can’t be managed with conservative measures and requires a surgical intervention to address the underlying etiology. We believe the underlying cause of this fistula is inadvertent enterotomy during the process of fistula maturation. While peristomal fistulas have been described before with resultant abscess formation, chronic peristomal fistulas inducing pseudoverrucous lesions are unique and can create a diagnostic challenge [13]. One can postulate the chronic chemical irritation of the skin facilitates PEH growth as a protective mechanism. Initial management should include local skincare, topical anti-inflammatory ointments, and attention to the fitting and pouching technique. If this doesn’t resolve with conservative measures, suspicion should be made to a propagating factor. Our patient had an ongoing small peristomal fistula that was controlled enough not to present with overt observable output. That fistula created this constant chemical irritation that drove PEH growth in our patient. This is evident also in the gravity-dependent nature of his growth, being mostly concentrated at the lower border of his stoma. In resistant cases, like this one, the surgical approach should focus on assessing for underlying etiology and ruling out any associated malignancy more than the mere attempt of removing the lesion to alleviate symptoms.

## Conclusions

Pseudoepitheliomatous hyperplasia is a benign skin condition characterized by hyperplastic growth of the epidermis. It can be related to infectious, neoplastic, and inflammatory conditions. Adequate management of peristomal pseudoepitheliomatous hyperplasia starts with local skincare and meticulous placement of the stoma appliance. This novel report illustrates the role of surgical resection in resistant cases to alleviate symptoms and assess for underlying etiologies, such as an apparent fistula, as well as to rule out any neoplastic growth.
